# LncRNA HOXA‐AS3 promotes the malignancy of glioblastoma through regulating miR‐455‐5p/USP3 axis

**DOI:** 10.1111/jcmm.15788

**Published:** 2020-09-11

**Authors:** Wanghao Chen, Qiaoyu Li, Guilong Zhang, Hong Wang, Zhihan Zhu, Lukui Chen

**Affiliations:** ^1^ Medical School of Southeast University Nanjing China; ^2^ Department of Neurosurgery Affiliated People's Hospital of Jiangsu University Zhenjiang China; ^3^ Department of Neurosurgery Neuroscience Center Cancer Center Integrated Hospital of Traditional Chinese Medicine Southern Medical University Guangzhou China

**Keywords:** competing endogenous RNA, glioblastoma, lncRNA HOXA‐AS3, miR‐455‐5p, USP3

## Abstract

Our objective was to determine the molecular mechanisms by which lncRNA HOXA‐AS3 regulates the biological behaviour of glioblastoma multiforme (GBM). We used an lncRNA microarray assay to identify GBM‐related lncRNA expression profiles. Qrt‐PCR was used to survey the levels of expression of long non‐coding RNA (lncRNA) HOXA‐AS3 and the target gene. Dual‐luciferase reporter assays were used to investigate the interaction of lncRNA HOXA‐AS3, the target gene and miRNA. Western blot analysis was used to examine the expression of USP3 and epithelial‐mesenchymal transition (EMT) genes. The MTT assay, transwell assay and wound healing assay were used to analyse the effects of lncRNA HOXA‐AS3 on GBM cell viability, mobility and invasiveness, respectively. Our results showed that lncRNA HOXA‐AS3 was significantly up‐regulated in GBM cells and could promote GBM cell proliferation, invasion and migration in vitro and in vivo. HOXA‐AS was found to be associated with poor survival prognosis in glioma patients. The dual‐luciferase reporter assay also revealed that lncRNA HOXA‐AS3 acts as a mir‐455‐5p sponge by up‐regulating USP3 expression to promote GBM progression. Western blot analysis showed that lncRNA HOXA‐AS3 could up‐regulate EMT‐related gene expression in GBM. Experiments showed mir‐455‐5p could rescue the effect of lncRNA HOXA‐AS3 on cell proliferation and invasion. The newly identified HOXA‐AS3/mir‐455‐5p/USP3 pathway offers important clues to understanding the key mechanisms underlying the action of lncRNA HOXA‐AS3 in glioblastoma.

## 1 INTRODUCTION

Glioblastoma multiforme (GBM) is a fast‐growing type of aggressive and lethal primary brain tumour.^1^, ^2^ Because the pathologic mechanisms of GBM are not fully understood, more research is needed to identify effective predictive biomarkers and therapeutic targets for GBM.^2^, ^3^

Emerging studies, supported by whole‐genome sequencing technology and microarray assays, have shown long non‐coding RNAs (lncRNAs) to be key regulatory transcripts.^4^ LncRNAs are classified as RNAs if they are longer than 200 nucleotides. Most lncRNAs are poorly annotated coding genes.^5^ The expression of some lncRNAs is correlated with poor cancer prognosis.^6^, ^7^ LncRNAs regulate tumorigenesis and invasion by activating or silencing oncogenes via various mechanisms, such as epigenetic regulation, RNA decay and post‐translational modification.^8^ Many lncRNAs have high levels of expression in brain tumours such as GBM and astrocytoma, and these lncRNAs have been shown to rely on various molecular mechanisms.^9^

In the current study, we identified a novel lncRNA, HOXA cluster antisense RNA 3 (HOXA‐AS3). It was found to be one of the most up‐regulated lncRNAs in GBM tissues.^10^, ^11^ HOXA‐AS3 is a HOX cluster, and it is a set of transcription factor genes that regulate hematopoietic lineage differentiation and embryological development.^10^, ^12^ Members of the HOX cluster family, such as HOXA‐AS2, have been shown to regulate cell proliferation and the formation of tumour‐related vasculogenic mimicry in various tumours.^13^, ^14^ In contrast, there have been very few studies on the biological mechanism underlying the action of lncRNA HOXA‐AS3 in tumours.

We here identified lncRNA HOXA‐AS3 and observed characteristic up‐regulation of its expression in malignant glioma tissue relative to normal brain tissues, which is consistent with the information in the TCGA database. Down‐regulation of lncRNA HOXA‐AS3 has been shown to significantly suppress glioblastoma cell (LN229 and U251) proliferation, invasion and migration. We also found that lncRNA HOXA‐AS3 could act as a miRNA sponge to reduce miR‐455‐5p concentration, up‐regulate USP3 expression and drive the epithelial‐mesenchymal transition (EMT) process of GBM cells. This study is the first to provide evidence of a positive correlation between lncRNA HOXA‐AS3 and USP3. This study improved our understanding of the HOXA‐AS3/miR‐455‐5p/USP3 signalling pathway and may facilitate future development of new treatments for glioblastoma.

## 2 MATERIALS AND METHODS

### 2.1 TCGA data analysis

The processed lncRNA expression data of 502 cases of GBM and five cases of normal brain tissues were download from the TCGA database (http://cancergenome.nih.gov/).

### 2.2 Tumour tissues samples

Malignant glioma specimens (n = 25) and normal brain tissues (NBT; n = 10) were purchased from January 2009 to December 2019 at the People's Hospital affiliated with Jiangsu University. This study protocol was approved by the ethics committee of Jiangsu University Affiliated People's Hospital, and the procedures were performed in accordance with the approved guidelines. All tumours were characterized by two pathologists.

### 2.3 Cell culture

Glioblastoma multiforme cell lines (U251, LN229, U87 and U138) were obtained from the American Type Culture Collection. T98G and SNB19 were purchased from the BeNa culture collection (BNCC), and normal human astrocytes (NHAs) were obtained from Sciencell Research Laboratories. All cells were cultured in Dulbecco's modified Eagle's medium (DMEM; Life Technologies) supplemented with 10% foetal bovine serum (FBS; Life Technologies) and maintained in a humidified atmosphere containing 5% CO_2_ at 37°C. Lentivirus transfection was purchased from GenePharma (Shanghai) and used to establish LN229 cell line labelled with green fluorescent protein (GFP).

### 2.4 RNA extraction and lncRNA microarray analysis

Total RNA was isolated using TRIzol reagent (Invitrogen). Qualified total RNA samples were amplified by cDNA according to the Low Input Quick Amp WT Labeling Kit (Agilent) and standard operating procedures (SOPs). The expression profile of lncRNA in five malignant glioma and five NBT were screened by using lncRNA microarray (Shanghai Biotechnology Corporation). Tissue sample preparation and microarray hybridization were separately performed according to manufacturer's recommendations. qRT‐PCR was used to further determine the nuclear/ cytoplasmic level of lncRNA HOXA‐AS3 in glioma cells. GAPDH and U6 were used as a nuclear and cytoplasmic control. The primer sequences for the amplification of the lncRNA HOXA‐AS3 and GAPDH used were listed in Table S1

### 2.5 Quantitative RT‐PCR (qRT‐PCR)

SYBR^TM^ Green PCR Master Mixs (ABI, USA) were designed for quantitative RT‐PCR. A LightCycler 490 Probes Master kit was also used to detect the relative expression of gene, and GAPDH was applied as the internal control for normalization. qRT‐PCR results were shown using the 2‐ΔΔCt method.

### 2.6 Oligonucleotide transfection

The small interfering RNA (siRNA) and vector were purchased from Shanghai Gene Pharmaceutical Company. These plasmids were transfected into LN229 and U251 cells with Lipofectamine 3000 (Invitrogen) to obtain lentivirus soups complying with the manufacturer's instructions. siRNA sequence was shown in Table S1

### 2.7 Inducible shRNA knock‐down

Short hairpin RNA (shRNA) targeting lncRNA HOXA‐AS3 (sh‐HOXA‐AS3) is used to silence lncRNA HOXA‐AS3 in vivo. Lentiviral particles were packaged into LN229 cells by co‐transfection with plasmids. All shRNA sequences were shown in Table S1.

### 2.8 MTT assays

Cell proliferation was evaluated with an MTT kit (Promega) complying with manufacturer's instructions. Thereafter, 3 × 10^3^ cells were seeded in each well of a 24‐well plate and maintained in medium containing 10% FBS for 2 weeks, during which time the medium was replaced with MTT reagent every 4 days. Cell proliferation and viability were measured with absorbance at 490 nm.

### 2.9 Cell invasion assays

Cell invasion was analysed using Transwell chambers (Corning). In total, 1 × 10^5^ cells were incubated with 0.3 mL serum‐free growth medium into the upper chamber of a fibronectin‐coated polycarbonate membrane embedded in a Transwell device and then incubated at 37°C for 4 hours. The medium supplemented with 10% FBS was applied to the lower chamber as a chemoattractant. After 48 hours, cells adhering to the bottom surface were stained with 0.2% crystal violet and manually calculated to determine the invasion index.

### 2.10 Cell migration assays

The scratch assay was used for the study of cell motility. After 24 hours, the cells were seeded on 6‐well plates at an initial density of 5 × 10^5^ in DMEM supplemented with 10% FBS. After the cells reached confluence, a scuffed wound was introduced with a sterile pipette tip into each well. The injuries were observed using phase contrast microscopy on an inverted microscope.

### 2.11 Dual‐Luciferase reporter assays

For luciferase reporter generation, the target genes of lncRNA HOXA‐AS3 were predicted by using bioinformatics analysis. USP3 3’UTR was cloned into pmirGLO‐dual‐luciferase reporter as described previously.^15^ Mutant and wild‐type plasmids were acquired to preventing their binding with miR‐455‐5p from GenePharma (Shanghai). The sequence of wild‐type lncRNA HOXA‐AS3, mutant lncRNA HOXA‐AS3, wild‐type USP3 (USP3‐Wt) and mutant USP3 (USP3‐Mut) was inserted into pmirGLO reporter vector. lncRNA HOXA‐AS3‐WT and lncRNA HOXA‐AS3‐MUT were co‐transfected into LN229 cells with negative control (miR‐NC), miR‐455‐5p screen and miR‐455‐5p inhibitors. After two days, the luminescent signals of Renilla firefly were calculated with the double luciferase assay system (Promega).

### 2.12 Western blotting

The proteins of the cultured GBM cells were extracted, separated by electrophoresis and transferred to PVDF membranes (Millipore). Protein concentrations were blocked with bovine serum albumin and then incubated with primary antibodies at 4°C overnight. The membranes were then incubated with secondary antibody at room temperature for one hour. Immunoreactivity was detected using the ECL chemiluminescent detection system (Thermo Fisher Scientific). USP3 and GAPDH antibodies (1:5000) were purchased from Thermo Fisher Scientific. Primary antibodies against E‐cadherin, N‐cadherin and vimentin (1:1000) were purchased from Abcam Biotechnology.

### 2.13 RNA pull‐down assay

A pull‐down test was used to examine the potential association of lncRNA HOXA‐AS3 with miRNA. The cells were treated and quantified with 1 mL of cellular nuclear lysate buffer for 3 days. Next, the cells were incubated with magnetic beads coated with streptavidin (Sigma) at 25°C for 2 hours, and the withdrawal test was carried out in an RNA complex coupled to biotin. After removal, the beads were washed in lysis buffer and RNA complexes bound to the beads were collected by centrifugation. The RNA in the beads was eluted and detected by qRT‐PCR.

### 2.14 Tumour xenograft model

Nude mice (4‐5 weeks old) were purchased from the Shanghai Center for Experimental Animals, Chinese Academy of Sciences. In the subcutaneous model, LN229 cells (1 × 10^6^) were injected subcutaneously into individual nude mice (n = 4/group). Tumour volume was measured once four day after injection. 32 days later, the mice were killed, and tumour weight was measured. Growth curves were displayed by plotting tumour volume against time. The tumour was removed and weighed, and then, RNA was extracted for qRT‐PCR. All of the animal experiments followed the experimental animal use guidelines of the National Institutes of Health. Then, LN229 cells were transfected with lentivirus sh‐HOXA‐AS3 in vitro for two days. Stereotactic implantation of sh‐HOXA‐AS3/LN229 cells was used to establish an intracranial xenotransplantation model. Mice were monitored by using IVIS Lumina II imaging system (Waltham, MA, USA) on 7 days and 21 days.

### 2.15 Statistical analysis

All statistical analyses were performed by SPSS 21.0 (IBM). Each experiment was repeated three or more times, as mentioned in each of the figure legends. Count data were expressed as a percentage or as a ratio, and the chi‐square test was applied for comparison. The results were analysed using Student's t test or unidirectional analysis of variance (ANOVA). Data represent mean ± SD. *P < .05, **P < .01 and ***P < .001 were considered statistically significant.

## 3 RESULTS

### 3.1 LncRNA HOXA‐AS3 expression was up‐regulated in GBM tissue and cell lines

**Figure 1:**
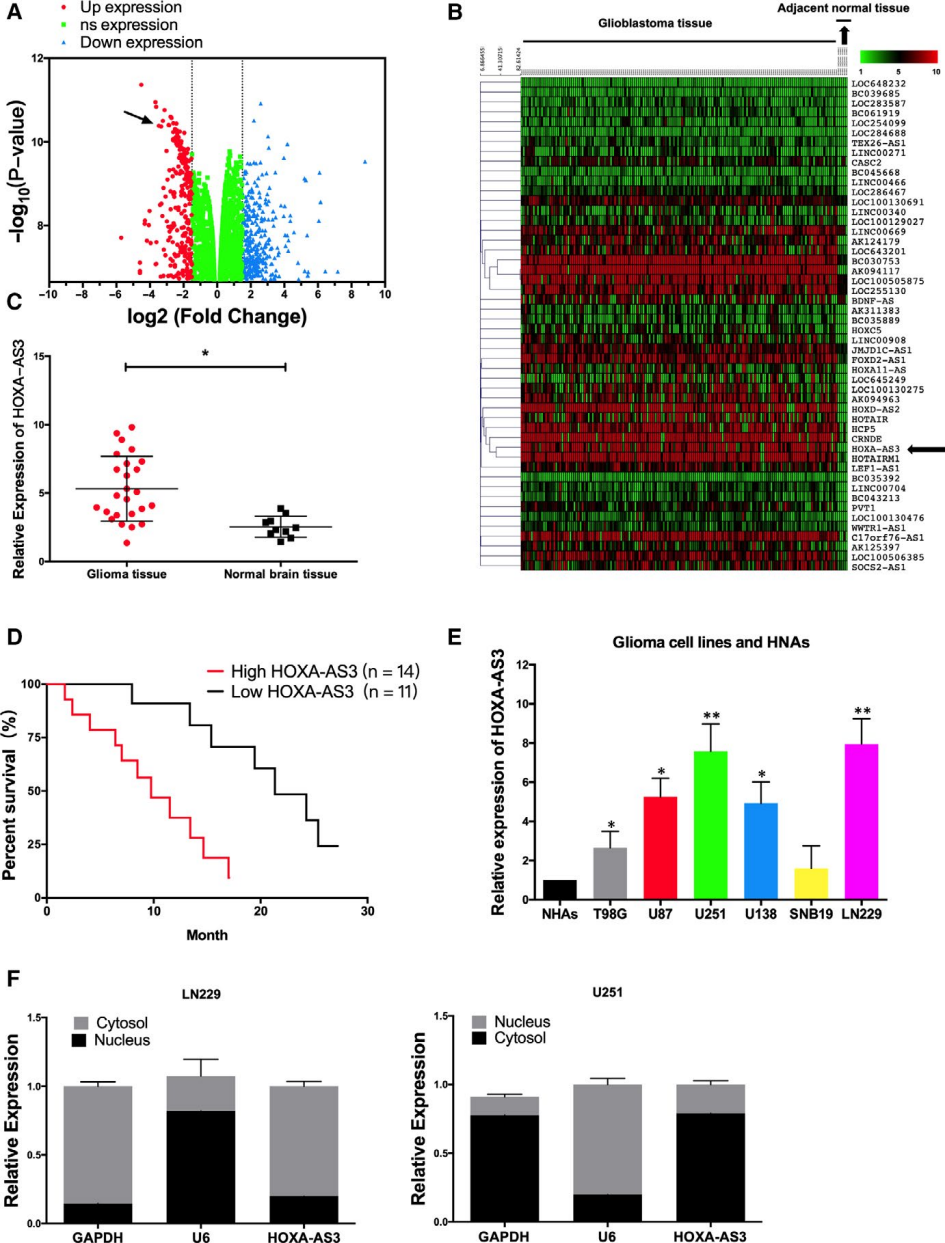
LncRNA HOXA‐AS3 was up‐regulated in glioblastoma patients and glioblastoma cells. A, Volcano plots show differentially expression levels of lncRNA in glioblastoma multiforme (GBM) tissues compared with normal tissues analysis. B, A heatmap is shown the expression of lncRNA for GBM samples (n = 502) compared to normal tissue samples (n = 5). Red represents lncRNAs that were up‐regulated in GBM. C, QRT‐PCR analysis of expression levels of lncRNA HOXA‐AS3 in GBM tissue and normal brain tissues (**P < .01). D, The survival rate between higher expression of lncRNA HOXA‐AS3 (n = 14) and lower expression of lncRNA HOXA‐AS3 (n = 11) in GBM patient was calculated by Kaplan‐Meier curve. E, QRT‐PCR analysis revealed lncRNA HOXA‐AS3 obviously up‐regulated in glioma cell lines compared with NHAs (*P < .05, **P < .01). F, lncRNA HOXA‐AS3 was mainly located in the cytoplasm of the GBM cells

LncRNA microarray assay was used to detect abnormal expression of thousands of lncRNAs in malignant glioma tissues (Figure 1A). LncRNA HOXA‐AS3 showed the most up‐regulated of 11.3‐fold change, which was selected as a candidate for further examination. In addition, the lncRNAs expression level of GBM patients from TCGA database showed that lncRNA HOXA‐AS3 was a significant up‐regulated lncRNA in GBM tissue. (Figure 1B). The expression of lncRNA HOXA‐AS3 in malignant glioma patients’ tissues and normal brain tissues was validated by RT‐PCR (Figure 1C). Then, Malignant glioma clinical samples were divided into two subsets with low expression versus high expression level. Kaplan‐Meier analysis showed that patients with higher levels of lncRNA HOXA‐AS3 had significantly poorer overall survival rates than those with lower expression of this lncRNA in GBM patients (Figure 1D). Furthermore, qRT‐PCR was used to assess the expression of lncRNA HOXA‐AS3 in various glioma cell lines such as U251, LN229, U87, T98G, SNB19 and U138. The expression of lncRNA HOXA‐AS3 was up‐regulated in GBM cell lines (U251, LN229, SNB19 and U138) compared with normal cell lines (NHA) (Figure 1E). The LN229 and U251 cell lines were selected for the other experiments because lncRNA HOXA‐AS3 had higher expression relative to the other GBM cell lines. LncRNA HOXA‐AS3 was found in the cytoplasm of LN229 and U251 cells, suggesting that lncRNA HOXA‐AS3 may regulate the process of GBM in the nucleus (Figure 1F).

### 3.2 Knock‐down of lncRNA HOXA‐AS3 reduces GBM cell tumorigenicity and EMT process

**Figure 2:**
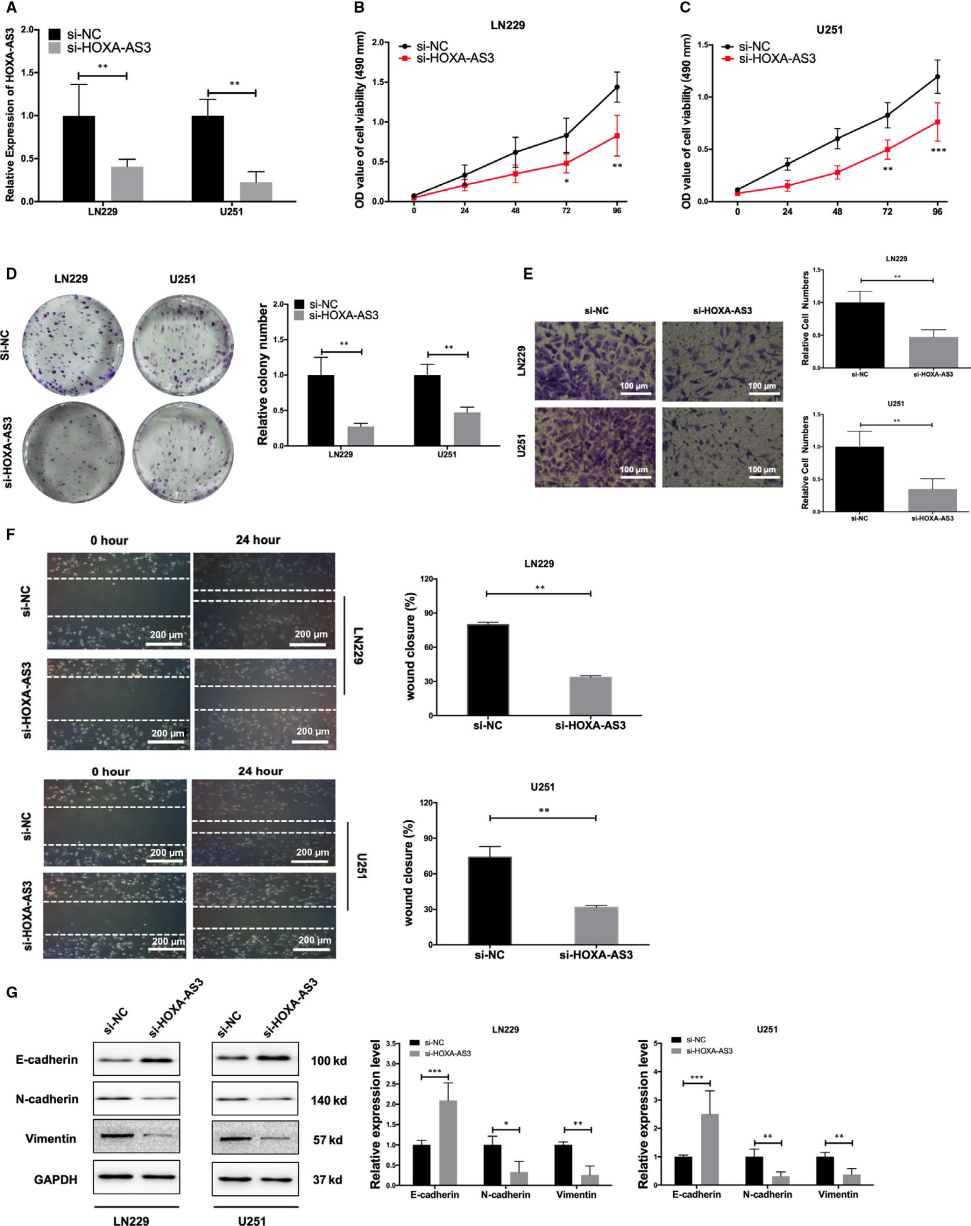
Knock‐down of lncRNA HOXA‐AS3 reduces glioblastoma multiforme cell tumorigenicity and EMT process in vitro. A, The efficiency of lncRNA HOXA‐AS3 knock‐down was detected by qRT‐PCR (**P < .01). B, MTT experiments suggested that lncRNA HOXA‐AS3 knock‐down attenuated the proliferative capacity of LN229 cells (**P < .01, ***P < .001). C, MTT experiments suggested that lncRNA HOXA‐AS3 knock‐down attenuated the proliferative capacity of U251 cells (**P < .01, ***P < .001). D, Colony formation assay manifested lncRNA HOXA‐AS3 knock‐down reduces LN229 and U251 cells tumorigenicity (**P < .01). E, Transwell experiments revealed knock‐down of lncRNA HOXA‐AS3 inhibited LN229 and U251 cell invasion (**P < .01). F, The wound healing assay showed a significant decrease of LN229 and U251 cells migration after transfected lncRNA HOXA‐AS3 inhibitor (**P < .01). G, Western blot detected the different expression levels of EMT‐related gene (E‐cadherin N‐cadherin and vimentin) in si‐HOXA‐AS3 group and blank control group glioma cells (**P < .01, ***P < .001)

To evaluate the functionality of lncRNA HOXA‐AS3 in vitro and in vivo, we reduced lncRNA HOXA‐AS3 expression in LN229 and U251 cells by using siRNA. The efficiency of lncRNA HOXA‐AS3 down‐regulation was tested by qRT‐PCR (Figure 2A). Functionally, colony formation and MTT assay showed that lncRNA HOXA‐AS3 lncRNA degradation significantly reduced cell proliferation for LN229 and U251 cells after si‐HOXA‐AS3 transfection (Figure 2D). Using transwell assays, we demonstrated that silencing lncRNA HOXA‐AS3 could decrease invasion and migration functions of GBM cells (Figure 2E). We also found that knock‐down of lncRNA HOXA‐AS3 expression strongly inhibited flattening and spreading in a cell‐wounding assay, suggesting that lncRNA HOXA‐AS3 expression dramatically affects key tumorigenesis gene signatures (Figure 2F). Western blotting analysis was performed to verify EMT‐related biomarkers (E‐cadherin, N‐cadherin and vimentin) expression. These results showed knock‐down of lncRNA HOXA‐AS3 can increase E‐cadherin protein expression and reduce inhibit N‐cadherin and vimentin protein expression (Figure 2G).

### 3.3 Overexpression of lncRNA HOXA‐AS3 promotes glioblastoma cell proliferation and invasion

To assess whether lncRNA HOXA‐AS3 can also promote glioblastoma cell proliferation, we used a simulator to regulate lncRNA HOXA‐AS3 expression in LN229 cells and U251 cells. By transfecting cells with a vector carrying lncRNA HOXA‐AS3, we constructed a stable lncRNA HOXA‐AS3 overexpressing cell line. The efficiency of lncRNA HOXA‐AS3 overexpression was detected by qRT‐PCR (Figure S1A). Next, MTT and transwell examinations were performed to determine the effect on cell viability and invasion (Figure S1B‐D). Cells transfected with lncRNA HOXA‐AS3 showed significant cell proliferation enhancement compared with cells transfected with blank control. In addition, wound healing experiments showed that lncRNA HOXA‐AS3 overexpression significantly promoted the migration capacity in two GBM cell lines (Figure S1E‐F). These data suggest that lncRNA HOXA‐AS3 overexpression may enhance glioblastoma cell proliferation and invasion in vitro.

### 3.4 Deregulation of lncRNA HOXA‐AS3 suppresses GBM cell proliferation and invasion in GBM orthotopic xenografts

**Figure 3:**
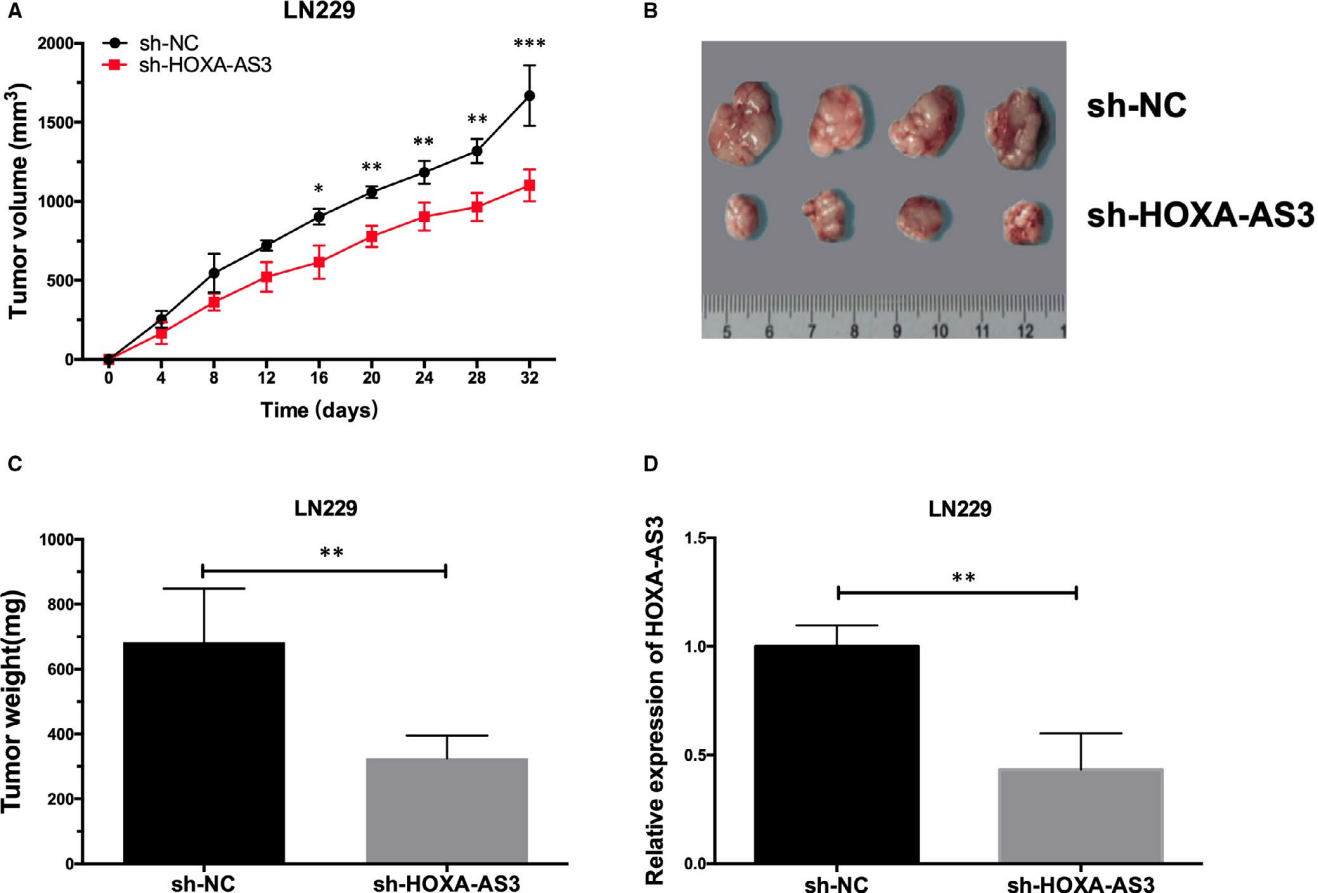
LncRNA HOXA‐AS3 depression inhibits glioblastoma tumorigenesis in vivo. A, The volume tumours formed by the LN229/sh‐HOXA‐AS3 cells were significantly smaller relative to those formed by the LN229/sh‐NC cells at the end of the experiment (*P < .05, **P < .01 and ***P < .001). B, Images of dissected tumours after the sh‐NC cells and sh‐HOXA‐AS3 cells were subcutaneous injection into the two groups of nude mice. C, The average weight significantly differed between the two groups (**P < .01). D, lncRNA HOXA‐AS3 expression of LN229/sh‐HOXA‐AS3 group was significantly lower relative to LN229/sh‐NC group in tumour tissues (**P < .01)

To further evaluate the potential therapeutic value of lncRNA HOXA‐AS3 inhibition in vivo, we transfected shRNA lncRNA HOXA‐AS3 plasmid vectors into LN229 cells (sh‐HOXA‐AS3/LN229) for orthotopic GBM xenografts. Then, we injected nude mice subcutaneously with sh‐HOXA‐AS3/LN229 cells and measured the tumour volume every 4 days thereafter for 32 days. The quantification of the tumour volume showed that tumour growth was slowed down in the mice injected with sh‐HOXA‐AS3 cells compared to the sh‐NC cells (*P* < .01) (Figure 3A,B). As shown in Figure 3C, treatment with sh‐HOXA‐AS3 also resulted in a significant reduction in tumour weight. We further extracted RNA from tumour tissues for qRT‐PCR analysis, and the results showed that the expression of lncRNA HOXA‐AS3 in sh‐HOXA‐AS3 tissues was significantly lower relative to control tissues (Figure 3D). These results suggest that suppression of lncRNA HOXA‐AS3 may have therapeutic potential for established tumours.

### 3.5 LncRNA HOXA‐AS3 acts as a miRNA sponge and negatively regulates miR‐455‐5p expression

**Figure 4:**
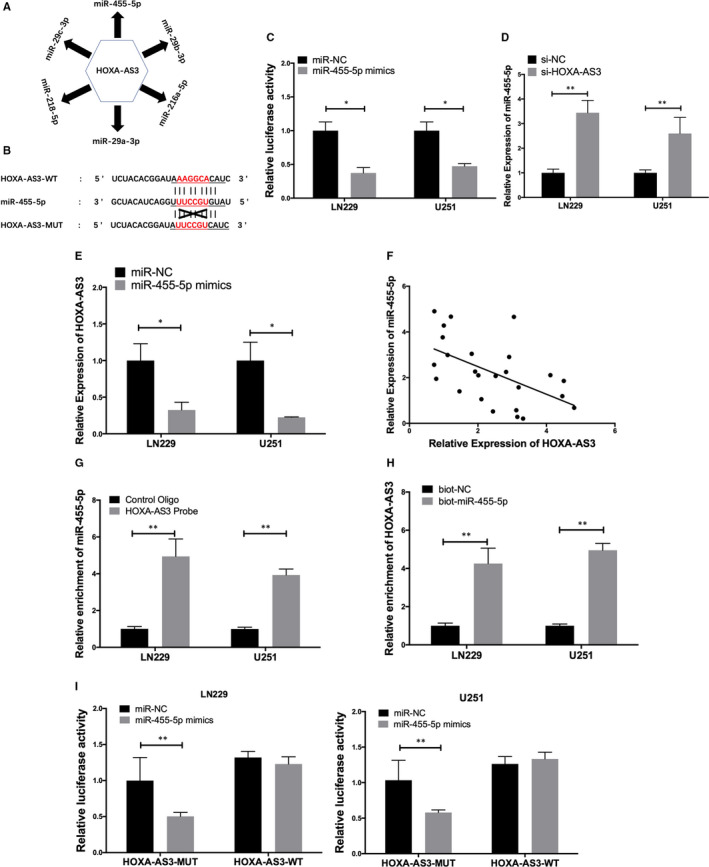
LncRNA HOXA‐AS3 acts as a miRNA sponge and negatively regulates miR‐455‐5p expression. A, Network diagram illustrates the regulatory correlation between lncRNA and miRNA based on the data from the bioinformatics software (Starbase v2.0). B, Schematic representation of the wild‐type or mutant miR‐455‐5p binding sequence predicted in lncRNA HOXA‐AS3. C, Luciferase activity analysis showed that lncRNA HOXA‐AS3 expression in miR‐455‐5p‐overexpression group or blank control group (*P < .05). D, qRT‐PCR revealed that knock‐down of lncRNA HOXA‐AS3 significantly up‐regulates miR‐455‐5p expression in LN229 and U251 cells (**P < .01). E, qRT‐PCR revealed that ectopic expression of miR‐455‐5p decrease lncRNA HOXA‐AS3 level in LN229 and U251 cells (*P < .05). F, The negative correlation between lncRNA HOXA‐AS3 and miR‐455‐5p in glioblastoma multiforme (GBM) tissues. G, The expression level of lncRNA HOXA‐AS3 was detected by qRT‐PCR after pull‐down (**P < .01). H, The expression level of miR‐455‐5p was assayed by qRT‐PCR after pull‐down (**P < .01). I, Co‐transfection of lncRNA HOXA‐AS3 3'‐UTR wild‐type or mutant seed region constructed with Over‐miR‐455‐5p in GBM cells (**P < .01)

Accumulating evidence indicates that lncRNAs serve as a sponge to negatively regulate certain miRNAs.^16^, ^17^ To investigate whether lncRNA HOXA‐AS3 utilizes a similar mechanism, bioinformatics software (StarBase v2.0) was used to identify lncRNA HOXA‐AS3 binding sites (Figure 4A,B). Luciferase activity analysis showed that compared with the blank vector control, the lncRNA HOXA‐AS3 level of miR‐455‐5p overexpression group was significantly reduced by 51% (Figure 4C). In addition, qRT‐PCR showed that knock‐down of lncRNA HOXA‐AS3 significantly up‐regulated the expression of miR‐455‐5p in LN229 and U251 cells (Figure 4D). Meanwhile, qRT‐PCR showed that ectopic expression of miR‐455‐5p reduced the expression level of lncRNA HOXA‐AS3 in LN229 and U251 cells (Figure 4E). We used qRT‐PCR to reveal a negative correlation between lncRNA HOXA‐AS3 and miR‐455‐5p expression in malignant glioma tissue (Figure 4F). To further identify miR‐455‐5p sponge lncRNA HOXA‐AS3, RNA pull‐down analysis was performed. The specific biotin‐labelled probe of lncRNA HOXA‐AS3 and control probe was designed for pull‐down assay and was confirmed the effect of the probe by qRT‐PCR (Figure 4G). We also used inverse pull‐down assay to test whether miR‐455‐5p could pull‐down lncRNA HOXA‐AS3 (Figure 4H). Luciferase reporter assay was also used to further prove the direct binding between lncRNA HOXA‐AS3 and miR‐455‐5p. Next, we constructed luciferase reporters containing lncRNA HOXA‐AS3 wild‐type binding sites (HOXA‐AS3 WT) or lncRNA HOXA‐AS3‐mutant binding sites (HOXA‐AS3 Mut). As expected, the luciferase activity of lncRNA HOXA‐AS3 WT was significantly inhibited by miR‐455‐5p in LN229 and U251 cells. In contrast, we did not observe any statistical change in the luciferase activity of lncRNA HOXA‐AS3 Mut (Figure 4I). These findings indicate that there is a direct interaction between miR‐455‐5p and lncRNA HOXA‐AS3, and miR‐455‐5p may be a promising miRNA for the lncRNA HOXA‐AS3 sponge in GBM.

### 3.6 USP3 is the target gene of miR‐455‐5p in GBM

**Figure 5:**
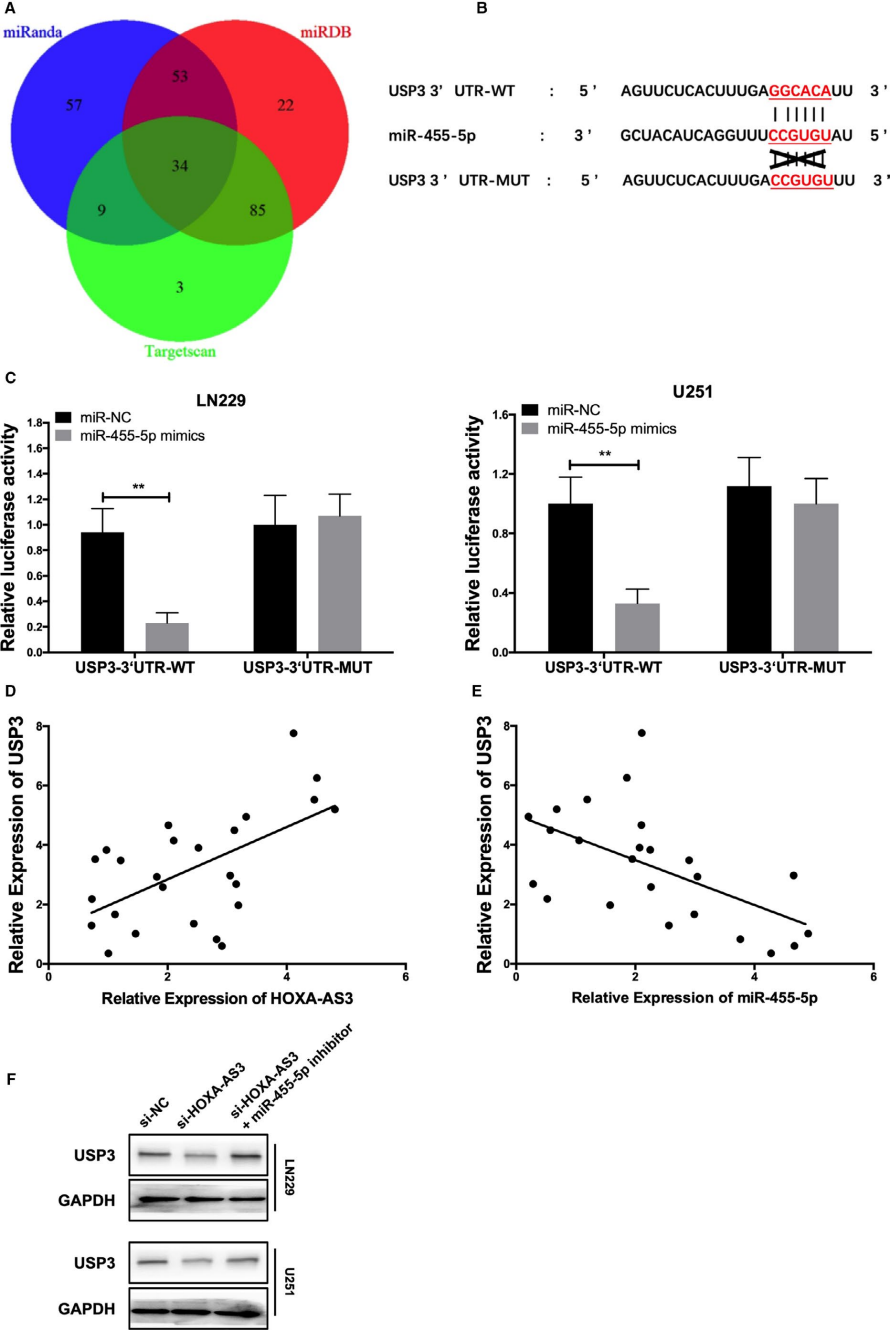
USP3 is the target gene of miR‐455‐5p in glioblastoma multiforme (GBM). A, The Venn diagram shows the miR‐455‐5p target gene predicted by miRDB (red), Targetscan (green) and miRanda (blue). B, According to the binding site information provided by Targetscan, design USP3‐MUT or USP3‐WT to explore the interaction between USP3 and miR‐455‐5p. C, The luciferase activities of USP3‐WT and USP3‐MUT were measured in GBM cells transfected miR‐455‐5p mimics or miR‐NC with dual‐luciferase reporter assay (**P < .01). D, Pearson correlation showed the correlation between lncRNA HOXA‐AS3 and USP3 in GBM (**P < .01). E, Pearson correlation showed the correlation between miR‐455‐5p and USP3 in GBM. F, Effect of lncRNA HOXA‐AS3 and miR‐455‐5p interactions on USP3 expression was quantified by Western blot

To identify miR‐455‐5p‐mediated downstream regulators of cell growth and invasion in glioma, we applied three miRNA target prediction algorithms (Targetscan, miRDB and miRanda). We found 34 genes clustered in the aggressive and proliferation process (Figure 5A). Among the many miR‐455‐5p targets, we focused on ubiquitin specific protease 3 (USP3) for further investigation due to miR‐455‐5p could bind to the 3′‐UTR region of USP3 (Figure 5B). Therefore, we constructed USP3 3'UTR‐WT and USP3 3'UTR‐MUT. Both of plasmids were performed for luciferase reporter assay. These results showed that miR‐455‐5p mimic significantly inhibited the luciferase activity of USP3 3'UTR‐WT, while USP3 3'UTR‐MUT was not affected, suggesting that USP3 is a direct target of miR‐455‐5p (Figure 5C). We further found that USP3 expression was positively correlated with lncRNA HOXA‐AS3 expression and negatively correlated with miR‐455‐5p expression in glioma tissues (Figure 5D,E). At the same time, the effect of lncRNA HOXA‐AS3 and miR‐455‐5p interaction on USP3 expression was quantified by Western blot analysis (Figure 5F). These evidences indicate that lncRNA HOXA‐AS3 can regulate the expression of USP3 in glioma through miR‐455‐5p.

### 3.7 Overexpression miR‐455‐5p suppresses the effect of lncRNA HOXA‐AS3 on proliferation and invasion of GBM

**Figure 6:**
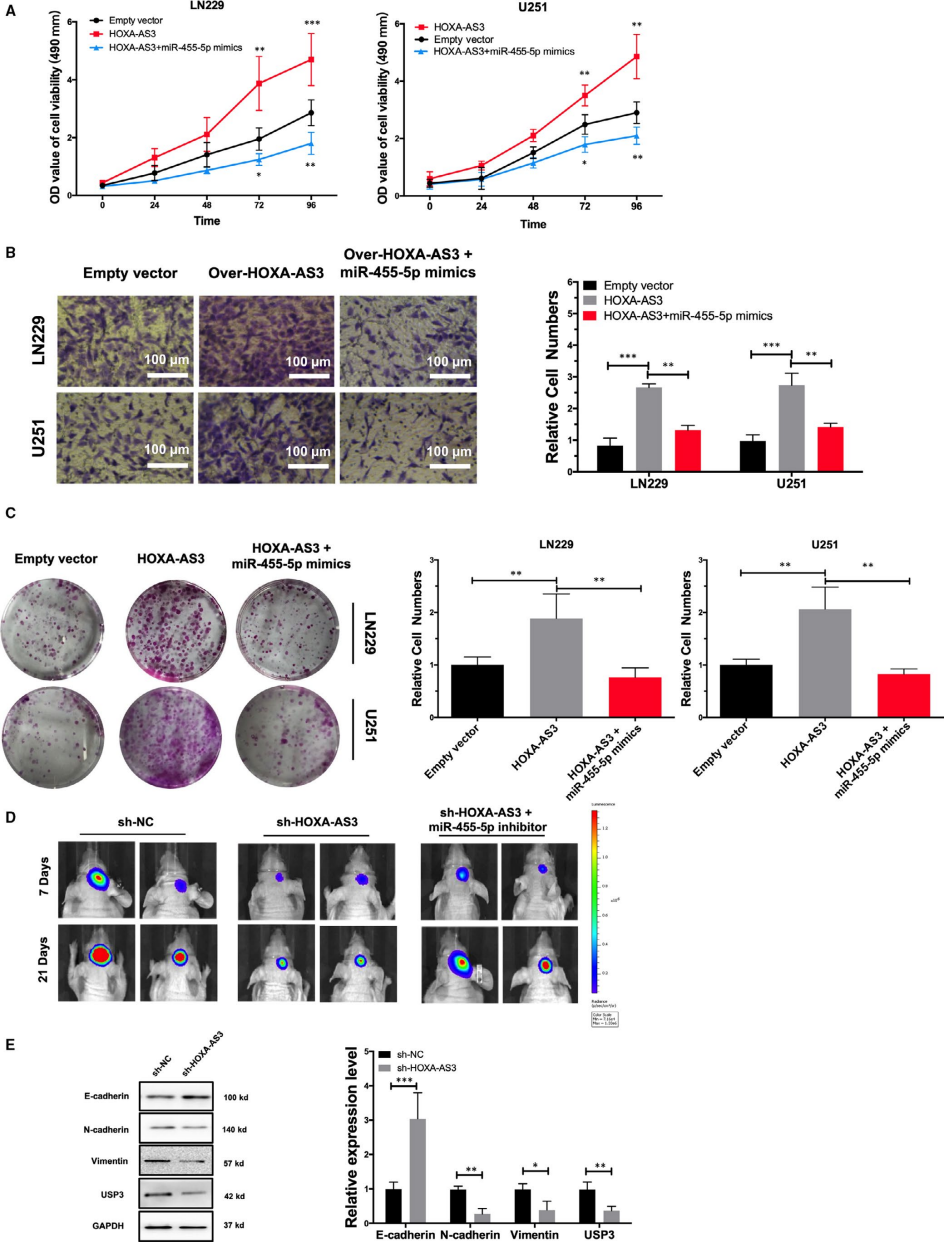
MiR‐455‐5p rescued effect on the proliferation and invasion of lncRNA HOXA‐AS3 overexpression glioblastoma multiforme (GBM) cells. A, MTT experiments suggested that MiR‐455‐5p rescued the effect of lncRNA HOXA‐AS3 on migration of LN229 cell and U251 cell (**P < .01, ***P < .001). B, Transwell assay was conducted to evaluate the effect of miR‐455‐5p on the proliferation of lncRNA HOXA‐AS3 overexpressed LN229 cells and U251 cells (**P < .01, ***P < .001). C, Colony formation assay showed that miR‐455‐5p inhibited the cell proliferative capacity rescued by lncRNA HOXA‐AS3 (**P < .01). D, Representative images of tumour viewed by IVIS after control group, shRNA lncRNA HOXA‐AS3 group and miR‐455‐5p inhibitor group inoculation. E, Western blot analysis showed protein expression of E‐cadherin, N‐cadherin, vimentin and USP3 in orthotopic xenograft tumour lncRNA HOXA‐AS3 knock‐down group and NC control group (*P < .05,**P < .01 and ***P < .001)

To validate the role of the lncRNA HOXA‐AS3/miR‐455‐5p/USP3 axis, a rescue test was used. The results showed that miR‐455‐5p reversed the effects of the lncRNA HOXA‐AS3 overexpression on GBM cell proliferation and invasion (Figure 6A‐C). To further confirm the interaction between miR‐455‐5p and lncRNA HOXA‐AS3, LN229 cells were labelled with luciferase expression and transfected with lncRNA HOXA‐AS3 shRNA. Then, the transfected cells were inoculated into the brain of nude mice. After 3 weeks, IVIS results showed that miR‐455‐5p inhibitor rescued the effect of the down‐regulation of lncRNA HOXA‐AS3 on the cell proliferation LN229 cells (Figure 6D). Previous studies have shown that USP3 raised GBM progression by regulating EMT.^18^ We explored the expression of EMT‐related genes in GBM orthotopic xenografts from different treatment groups. Western blot analysis showed that down‐regulation of lncRNA HOXA‐AS3 inhibits USP3 expression and EMT in vivo (Figure 6E).

## 4 DISCUSSION

Glioblastoma multiforme treatment is challenging and understanding the molecular mechanisms of malignant glioma is desirable in order to find a new therapy. Recent studies on lncRNA expression profiling have shown that lncRNA expression is significantly altered in glioma tissue, and many lncRNAs have shown as important players in cancer development and metastasis.^19^, ^20^, ^21^ However, the functional roles and molecular mechanisms of lncRNA HOXA‐AS3 in glioma progression remain largely unknown. In this study, we found that lncRNA HOXA‐AS3 is significantly up‐regulated in glioma tissue compared to normal adjacent tissue. In addition, lncRNA HOXA‐AS3 expression was positively associated with adverse prognosis of malignant glioma patients. Regarding its cellular function, we found that knock‐down of lncRNA HOXA‐AS3 inhibited the proliferation, invasion and migration of GBM cells in vitro. In contrast, these capacities of GBM cells were significantly enhanced when lncRNA HOXA‐AS3 was overexpressed. In addition, we have demonstrated that overexpression of lncRNA HOXA‐AS3 can enhance tumorigenesis in vivo. Taken together, these results indicated that lncRNA HOXA‐AS3 may play a critical role in the progression of glioma.

Recently, evidence suggests that lncRNA acts as a miRNA sponge or competing endogenous RNA (ceRNA) in the regulatory network.^17^, ^22^ miRNAs are a class of small non‐coding RNAs that regulate gene expression by being forced to locate at target sites in the 3 'non‐transferable region (UTR) of the mRNA, thereby controlling their translation and degradation in the cells. Multiple miRNAs take on critical functions in the development and progression of tumours by fine‐tuning multiple intracellular signalling pathways that mediate various biological functions such as cell proliferation and migration.^23^, ^24^ MiR‐455‐5p has been considered as either an oncogene or a tumour suppressor in certain types of tumour. For example, miR‐455‐5p acts as an oncogene in lung cancer.^25^ In contrast, miR‐455‐5p exerts tumour‐suppressive effects in prostate cancer.^26^ However, the role of miR‐455‐5p in GBM has not been revealed. Using bioinformatics analysis, we found that miR‐455‐5p may be a promising target miRNA for lncRNA HOXA‐AS3. To further validate the predictions, using qRT‐PCR, we revealed a negative correlation between lncRNA HOXA‐AS3 and miR‐455‐5p in LN229, U251 cells and GBM tissues. In addition, we demonstrated the direct binding of lncRNA HOXA‐AS3 and miR‐455‐5p using a dual‐luciferase reporting assay, suggesting that lncRNA HOXA‐AS3 promotes GBM progression by sponging miR‐455‐5p. Therefore, we predict that lncRNA HOXA‐AS3 can regulate GBM processes through sponge miR‐455‐5p. To validate this prediction, we performed a rescue experiment, and the results showed that miR‐455‐5p could significantly rescue the effect of lncRNA HOXA‐AS3 on GBM proliferation and metastasis. Using bioinformatics analysis, we found that USP3 may be a promising target gene for miR‐455‐5p. The dual‐luciferase report assay has shown that miR‐455‐5p can bind to the 3'‐UTR region of USP3. These results indicate that lncRNA HOXA‐AS3 can promote glioma progression by regulating the miR‐455‐5p/ USP3 axis.

Epithelial‐mesenchymal transition is a process in which epithelial cells are transformed into mesenchymal cells and acquire motile and invasive characteristics.^27^, ^28^ Recent studies have proven that USP3 promotes GBM progress by regulating the EMT process.^18^, ^29^ Therefore, in order to study the molecular mechanism of lncRNA HOXA‐AS3 promoting GBM transfer, we detected the level of EMT‐related biomarkers in GBM cells overexpressed by lncRNA HOXA‐AS3 by Western blot analysis. We found that depression of lncRNA HOXA‐AS3 significantly decreased the expression of EMT‐related protein, indicating that lncRNA HOXA‐AS3 can participate in GBM transfer by regulating HOXA‐AS3/miR‐455‐5p/ USP3 axis.

In conclusion, we found an associated lncRNA network that regulates the tumour cell development network, consisting of lncRNA HOXA‐AS3, miR‐455‐5p and USP3. Our research not only contributes to the intensive efforts to elucidate the mechanism of lncRNA HOXA‐AS3, but also specifically encourages the development of clinic‐based lncRNA anti‐glioma diagnostics and therapeutics.

## CONFLICT OF INTEREST

The authors confirm that there are no conflicts of interest.

## AUTHOR CONTRIBUTION

**Wanghao Chen:** Conceptualization (equal); Data curation (equal); Formal analysis (equal); Funding acquisition (equal); Investigation (equal); Methodology (lead); Project administration (equal); Resources (equal); Software (lead); Supervision (equal); Validation (lead); Visualization (lead); Writing‐original draft (lead); Writing‐review & editing (lead). **Qiaoyu Li:** Funding acquisition (supporting); Project administration (supporting); Writing‐review & editing (supporting). **Guilong Zhang:** Conceptualization; Data curation; Funding acquisition. **Hong Wang:** Data curation (supporting); Formal analysis (supporting); Funding acquisition (supporting); Visualization (supporting). **Zhihan Zhu:** Conceptualization (supporting); Funding acquisition (supporting); Investigation (supporting). **Lukui Chen:** Conceptualization (equal); Data curation (equal); Formal analysis (equal); Funding acquisition (equal); Investigation (lead); Methodology (lead); Project administration (lead); Resources (lead); Software (lead); Supervision (lead); Validation (lead); Visualization (lead); Writing‐original draft (lead); Writing‐review & editing (lead).

## Supporting information

Figure S1

Table S1

## Data Availability

The data sets used or analysed in this study may be obtained from appropriate authors upon reasonable request.
