# Supplementary figures and images for: LncRNA HOXA‐AS3 promotes the malignancy of glioblastoma through regulating miR‐455‐5p/USP3 axis
Source: J Cell Mol Med. 2020 Sep 11;24(20):11755–67. doi: 10.1111/jcmm.15788 (PMC7579690; doi:10.1111/jcmm.15788)

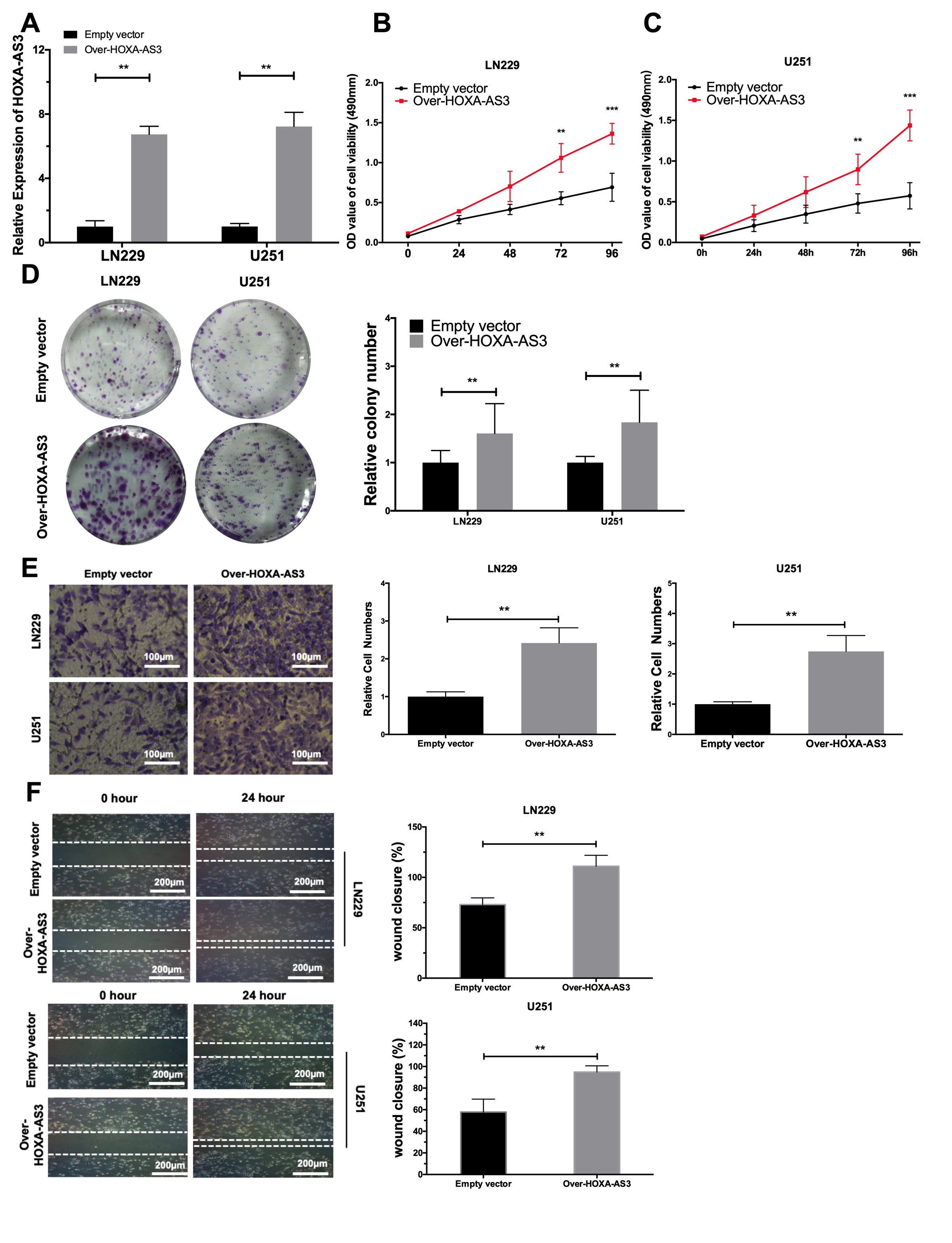

Supplement: Supplementary file 1 — Figure S1 [file JCMM-24-11755-s001.tiff]
