# Supplementary material for: LncRNA HOXA‐AS3 promotes the malignancy of glioblastoma through regulating miR‐455‐5p/USP3 axis
Source: J Cell Mol Med. 2020 Sep 11;24(20):11755–67. doi: 10.1111/jcmm.15788 (PMC7579690; doi:10.1111/jcmm.15788)
Supplement: Supplementary file 2 — Table S1 [file JCMM-24-11755-s002.docx]

**Supplement Table 1**

**The primer sequences of gene-specific primers used for real-time RT-PCR:**

HoxA-AS3 Forward: 5′-CACCTCTCTCATCGAAAAACCG-3′,

HoxA-AS3 Reverse: 5′- GCACCAGGAAAGAGGACAATTC-3′;

GAPDH Forward: 5′-TCGCTCCTGGAAGATGGTGAT-3′;

GAPDH Reverse: 5′-TCATTGACCTCAACTACATG-3′.

**The primer Sequences of siRNA or shRNA:**

shRNA-HOXA-AS3 sence: GGGCCGAACAACUCAUAAATT；

shRNA-HOXA-AS3 antisence: UUUAUGAGUUGUUCGGCCCTT.
